# Electronic structure and bandgap of γ-Al_2_O_3_ compound using mBJ exchange potential

**DOI:** 10.1186/1556-276X-7-488

**Published:** 2012-08-31

**Authors:** Mohsen Yazdanmehr, Saeid Jalali Asadabadi, Abolghasem Nourmohammadi, Majid Ghasemzadeh, Mahmood Rezvanian

**Affiliations:** 1 Department of Physics, Faculty of Science, University of Isfahan (UI), Hezar Gerib Avenue, Isfahan, 81744, Iran

**Keywords:** Bandgap, mBJ exchange potential, Density functional theory

## Abstract

γ-Al_2_O_3_ is a porous metal oxide and described as a defective spinel with some cationic vacancies. In this work, we calculate the electronic density of states and band structure for the bulk of this material. The calculations are performed within the density functional theory using the full potential augmented plan waves plus local orbital method, as embodied in the WIEN2k code. We show that the modified Becke-Johnson exchange potential, as a semi-local method, can predict the bandgap in better agreement with the experiment even compared to the accurate but much more expensive green function method. Moreover, our electronic structure analysis indicates that the character of the valence band maximum mainly originates from the p orbital of those oxygen atoms that are close to the vacancy. The charge density results show that the polarization of the oxygen electron cloud is directed toward aluminum cations, which cause Al and O atoms to be tightly connected by a strong dipole bond.

## Background

γ-Al_2_O_3_ is an important material in nanotechnology because of its porous structure, high surface area, and high catalytic surface activity. This material is widely used as a catalyst, an adsorbent, and a support for industrial catalysts in the oxidation of organics and the catalytic reduction of automotive pollutants such as nitric oxide [1]. This oxide material is a metastable ‘transition’ phase of aluminum oxide or alumina which can be formed via dehydrating boehmite γ-AlOOH at rather low temperatures (350°C to 1,000°C) [2] and is able to keep its crystalline structure unchanged up to about 1,200°C [3]. Recently, this material is considered as a suitable alternative to silicon for producing semiconductor nonvolatile random access memories for future applications [4]. Thus, investigation of the atomic and electronic structures of γ-Al_2_O_3_ has also received much attention over the last decade [5,6].

The density functional theory (DFT)-based methods are appropriate to investigate the electronic structure of solid materials. However, the precise electronic bandgap prediction is a shortcoming in the DFT-based methods (see Table 1).

**Table 1:** Bandgaps

**References**	**Method**	**Bandgap (eV)**
[6]	GGA	4.0
[14]	LDA	4.2
	PW91-GGA	3.8
[13]	G_0_W_0_	7.2
Current	GGA	4.13
	Regular mBJ	6.19
	Non-regular mBJ	8.02
Exp. [12]	-	8.7

*GGA*, generalized gradient approximation; *LDA*, local density approximation; *GW*, Green function; *mBJ*, modified Becke-Johnson.

In this work, we have calculated the electronic structure of γ-Al_2_O_3_ compound using the mBJ exchange potential [7]. Our result shows that the semi-local mBJ method predicts the γ-Al_2_O_3_ bandgap better than the LDA and GGA when compared to the experimental data. The results which are obtained by the semi-local mBJ method are in good agreement with those of the experimental data. In fact, the precision of theoretical predictions in the mBJ scheme is comparable with the accurate but much more expensive Green function (GW) method. In the mBJ exchange potential, there is a correction (*c*)-factor which can be used as an adjustable parameter for improving the bandgap prediction. Therefore, the mBJ method has the capability to overcome the well-known shortcoming of the DFT-based methods in predicting the bandgaps. In this paper, we have utilized this capability to yield the bandgap of γ-Al_2_O_3_ by determining and fixing the adjustable *c* parameter of the mBJ potential. The calculated bandgap within our fixed mBJ calculations is found to be in better agreement with that of the experiment than that of the GW method.

## Main text

### Methods

All of the calculations in this work were carried out using the WIEN2K code [8], which is based on the full potential augmented plane waves plus local orbital method. For the calculations, 96 irreducible points in the first Brillouin zone were used. The points were separated for integration over the Brillouin zone of the unit cell using a set of 8 × 8 × 3 special k-points. The expansion of the wave functions and charge densities was cut off by setting the R_MT_K_Max_, G_Max_, and Muffin-tin radii, as tabulated in Table 2. In this research, we have used the mBJ exchange potential to investigate the electronic structure and bandgap of bulk γ-Al_2_O_3_. This functional has been proposed by Tran and Blaha [7]. It is a modified version of the BJ exchange correlation potential (introduced by Becke and Johnson [9]) to improve the bandgap prediction of the DFT-based methods. In the regular mBJ calculations, we are only allowed to determine the *c*-factor self-consistently through our *ab initio* calculations. It is known that the mBJ potential cannot always successfully predict the bandgaps for all of the materials in the world in excellent agreement with the experiment [7]. However, it may be possible to efficiently improve the bandgap perdition by performing non-regular mBJ calculations. The *c*-factor is self-consistently determined in the regular mBJ calculations. In the non-regular mBJ calculation, one can internally increase the *c* parameter of the mBJ method to obtain better results. It has been shown that the bandgaps increase as the *c* parameter increases [7,10]. Thus, one can perform a non-regular mBJ calculation by increasing the *c* parameter to an optimized c value. In order to more precisely determine the bandgap for the γ-Al_2_O_3_ compound, we optimize the *c*-factor and then adjust the *c*-factor manually to the optimized value in our non-regular mBJ calculations (see [10]). We show that the non-regular calculation can efficiently improve the bandgap.

**Table 2:** Computational parameters

**K-point meshes**	**R**_**MT**_**-K**_**MAX**_**(bohr ×** **Ry**^**1/2**^**)**	**Separation energy (Ry)**	**G**_**Max**_**(Ry**^**1/2**^**)**	**Muffin-tin radii (bohr)**
8 × 8 × 3	7	8	12	1.51

#### Crystal structure

The perfect crystalline structure of γ-Al_2_O_3_ is described as a defective spinel, denoted as ${□}_{2\frac{2}{3}}Al_{21\frac{1}{3}}$O_32_, (□ = *Al* vacancy) [5,11]. To understand this formula, we should consider a perfect spinel, such as the MgAl_2_O_4_ structure. However, due to stoichiometry, γ-Al_2_O_3_ is not a perfect spinel because its cation/anion ratio is less than a stoichiometric spinel structure (i.e., 24/32). Hence, $2\frac{2}{3}$ aluminum vacancies are formed in each cell. Aluminum vacancies can present both in tetrahedral and octahedral sites, as the cations are divided in tetrahedral and octahedral positions in a stoichiometric spinel. However, the lowest energy configuration of γ-Al_2_O_3_ occurs when all of the aluminum vacancies are in octahedral sites with the largest possible inter-distances. The space symmetry group of a perfect spinel, such as the MgAl_2_O_4_ structure, is $Fd3\bar{m}$. In this symmetry group, the primitive cell is a triclinic cell of 14 atoms, and hence, it is denoted as Mg_2_Al_4_O_8_. An ideal spinel unit is therefore constructed with the lattice constant 7.911 Å [5], and all of the Mg atoms are replaced by aluminum. The cubic unit cell contains three Al_6_O_8_ primitive cells on ‘top’ of each other, and as a result, the unit cell contains 18 Al and 24 O atoms. To get the correct stoichiometry, we subsequently remove two Al atoms from the octahedral sites which have the largest inter-distances. In this way, a 40-atom triclinic cell containing eight Al_2_O_3_ formula units and two *Al* vacancies is obtained. In order to prepare a hexagonal cell similar to the structure already reported by Pinto and co-workers [6], we change the basis vectors as a→'=b→,b→'=−b→,c→'=a→+b→−c→, where $\vec{a}=7.911\left(0\frac{1}{2}\frac{1}{2}\right),\vec{b}=7.911\left(\frac{1}{2}0\frac{1}{2}\right),\vec{c}=7.911\left(\frac{3}{2}\frac{3}{2}0\right)$ .

**Figure 1:**
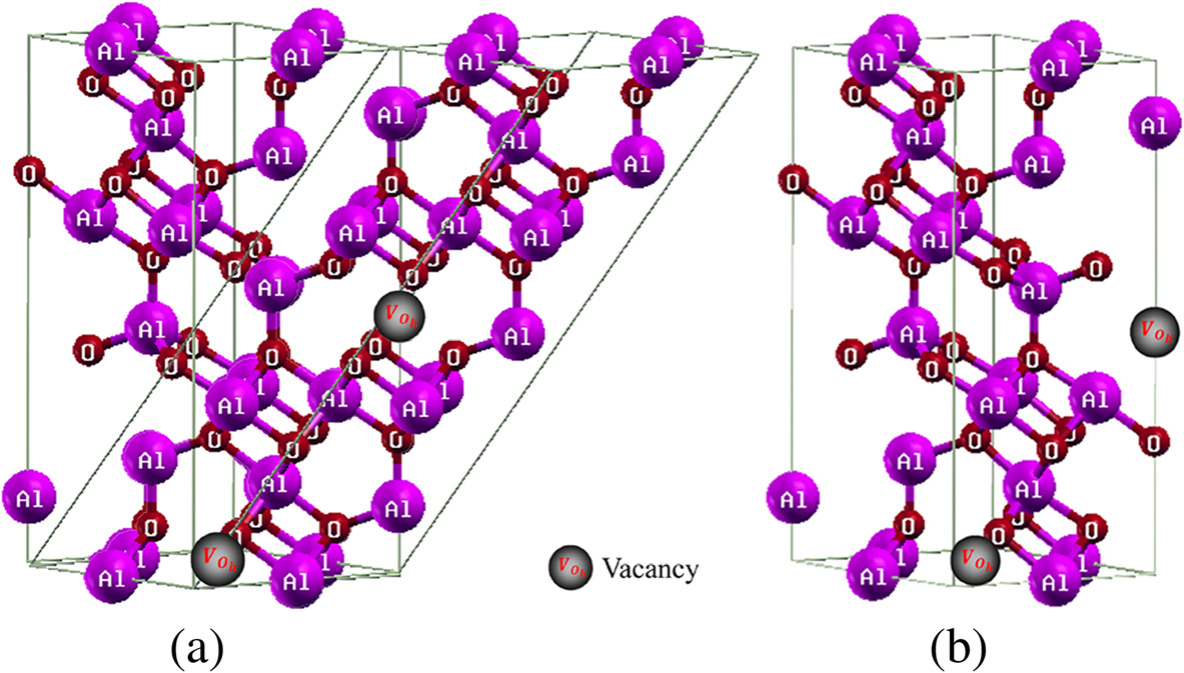
**Lattice unit cell conversion.** (**a**) Lattice unit cell conversion from triclinic to hexagonal. (**b**) Final γ-Al_2_O_3_ unit cell which is constructed in this study.

The prepared cell is then used for performing calculations. For a better illustration of the Al vacancy positions, a γ-Al_2_O_3_ supercell is constructed by doubling the size of the cell, as shown in Figure 1.

## Discussion

### Density of states

**Figure 2:**
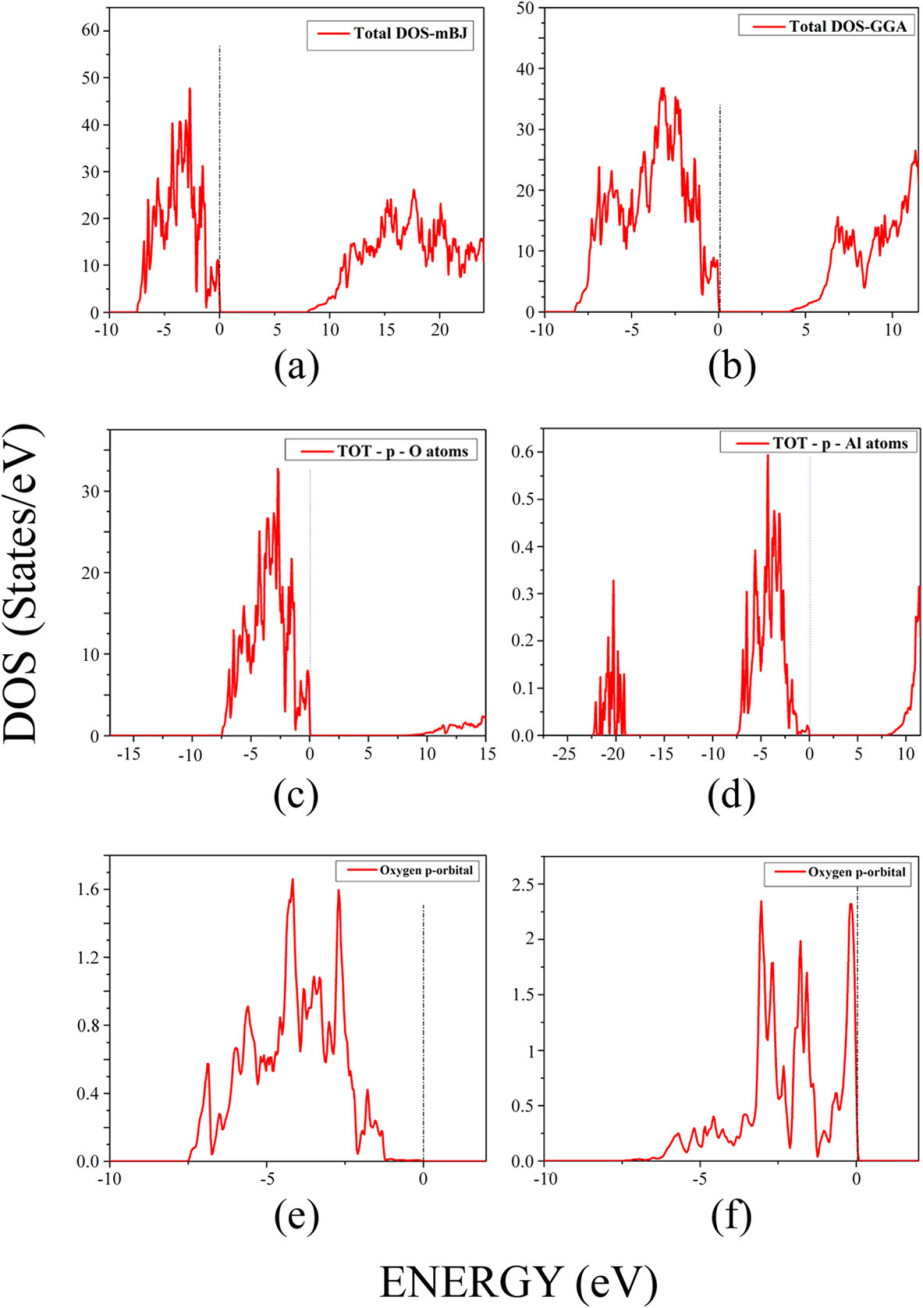
**Total and partial DOSs for γ-Al**_**2**_**O**_**3**_**.** Total DOSs for γ-Al_2_O_3_ by (**a**) mBJ and (**b**) GGA. Total partial p-DOSs within mBJ by adding all the p-DOSs of the nonequivalent (**c**) O atoms and (**d**) Al atoms, in the supercell shown in Figure 1b. Partial p-DOSs for those of O atoms which are (**e**) far from and (**f**) close to the vacancy. Fermi level is set to zero.

Total density of states (DOSs) is shown in Figure 2 within the mBJ (Figure 2a) and GGA (Figure 2b) for the defective spinel structure of γ-Al_2_O_3_. The total DOSs show that the mBJ causes the unoccupied states to move away from the Fermi level towards higher energies with respect to the conduction states calculated by the GGA. As can be seen from Figure 2a,b, the calculated occupied states within both the mBJ and GGA touch but do not cross the Fermi level. Therefore, according to the mBJ prediction, the valence and conduction states repel each other more strongly than that of the GGA. Hence, the stronger mBJ repulsion potential gives a larger bandgap than that of the GGA in more agreement with that of the experiment [12]. However, the predicted bandgap by the mBJ still underestimates the experimental value. We will discuss how to improve the bandgap prediction of the mBJ method in the subsequent section.

Partial p-orbital DoSs (partial p-DOSs) are calculated for each of the nonequivalent oxygen and aluminum atoms using mBJ in the γ-Al_2_O_3_ compound. The p-DOSs of the nonequivalent O atoms are added to each other to obtain total p-O-DOS, as shown in Figure 2c. Similarly, total p-DOS for the Al atoms is the sum over the partial p-DOSs of the nonequivalent Al atoms (Figure 2d). The total p-DOSs for O and Al atoms show that contribution of the oxygen p-states dominates valence states in the vicinity of the Fermi level. Therefore, most of the occupied states which are touching the Fermi level are constituted by the p-O-DOS. This implies that the oxygen atoms and, in particular, their p-sates are more important for tuning the band gap of γ-Al_2_O_3_. The latter result is deduced from the total p-DOS, obtained by adding p-DOSs of the nonequivalent O atoms. Hence, it is not still clear which of the oxygen atoms play a more important role. To clarify which of the oxygen atoms in the γ-Al_2_O_3_ compound are more appropriate for engineering the bandgap, we perform more elaborations on the electronic structure of the system by classifying the O atoms in two different groups. The first group contains the O atoms that are far from the vacancies. In the second group, the O atoms are selected to be close to the vacancies. The partial p-DOSs of the first and second groups are shown in Figure 2e,f, respectively. The results elucidate that the oxygen atoms in the second group play a more important role in the engineering of the bandgap, as a contribution of the valence p-DOS of those of oxygen atoms which are closer to the vacancies predominates in touching the Fermi level.

### Electronic structure and bandgap

**Figure 3:**
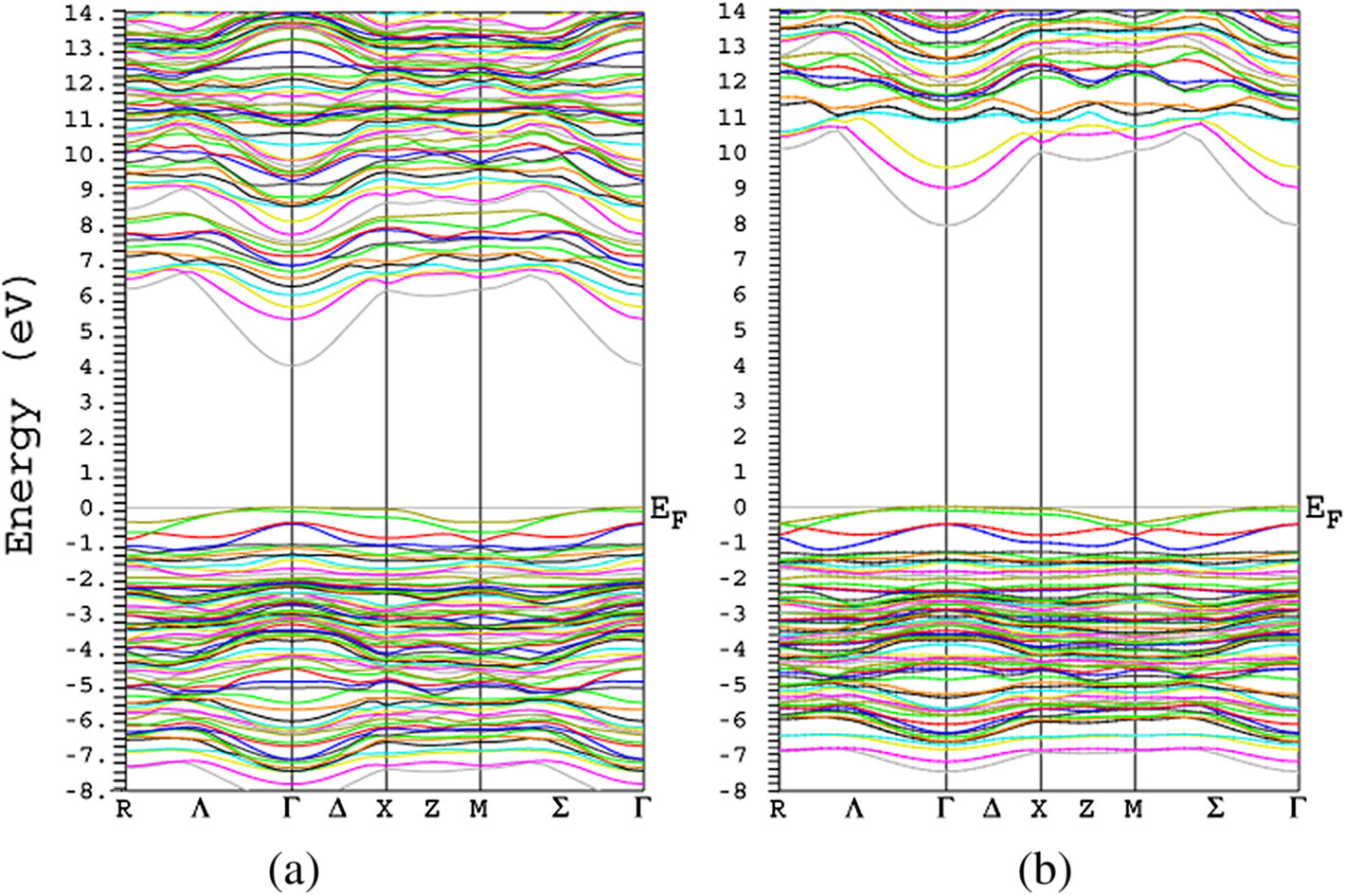
**Band structure calculated for γ-Al**_**2**_**O**_**3**_**.** Band structure using (**a**) GGA (**b**) and mBJ with *c* = 1.8.

Our calculation using the regular mBJ method yields a bandgap of 6.195 eV, as shown in Table 1. Although this value is much better than the previously calculated results obtained by the local and local-like density approximations, i.e., LDA or different versions of GGA, as can be seen from Table 1, it still underestimates the experimental value compared to that of the Green function method. In order to improve the bandgap prediction of the mBJ method, we calculate the band structure within a non-regular mBJ method (Figure 3b) in addition to the GGA band structure (Figure 3a) and the regular mBJ band structure. As can be seen from Figure 3b, the bandgap is significantly improved by our non-regular mBJ calculation compared to the GGA calculation (Figure 3a) and regular mBJ calculation (not shown), when compared to the experimental value of 8.7 eV [12]. More interestingly, our non-regular mBJ result (8.02 eV) is much better than that of the accurate G_0_W_0_ method (7.2 eV) [13], compared to the experimental value (8.7 eV) [12]. It is worth noting that the cost of GW calculation is more expensive than that of the mBJ since the mBJ is still a semi-local approximation. We now discuss the non-regular mBJ calculation. The regular mBJ prediction can be improved by gradually increasing the *c* parameter internally from its self-consistently converged value. The *c* parameter is converged to 1.45 for γ-Al_2_O_3_ within the regular mBJ calculation. However, this value is so small to be used for reproducing the experimental bandgap. Therefore, we increase the *c* parameter step by step. In this way, we optimize the *c*-factor of the mBJ method for our case. The optimized *c*-factor is found to be 1.8 for the γ-Al_2_O_3_ compound. In our non-regular mBJ calculations, we use this optimized value for the *c* parameter, keeping the parameter fixed during the whole of the self-consistent iterations, as discussed in [10]. This type of *c*-fixed mBJ calculation gives a direct bandgap of 8.02 eV at the Γ point, which is closer to the experimentally measured value of 8.7 eV [12] compared with those of both the regular mBJ and GW methods.

The bandgap of the system is improved by performing the non-regular mBJ calculation. However, it is mandatory to examine whether the internal structure of bands is destroyed by increasing the strength of the repulsion mBJ potential. To this end, one needs to compare Figure 3a and Figure 3b. The comparison authenticates that the band structure is not internally affected by the stronger repulsion potential. Indeed, the conduction bands are altogether shifted up by the same value after increasing the *c* parameter, keeping their previous structures almost the same as before. This is consistent with our discussion presented earlier concerning the behaviors of the regular mBJ- and GGA-DOSs, as can be rechecked by comparing Figure 2a and Figure 2b.

### Physical backbone of increasing the *c*-factor for the defective spinel γ-Al_2_O_3_

In order to physically support our success in predicting the bandgap within the non-regular mBJ calculations in better agreement with that of the experiment than that of the GW prediction, as presented in the preceding subsection, we attempt to discuss the physical mechanism underlying such an agreement in this subsection. In fact, the bandgap is well reproduced for the defective spinel γ-Al_2_O_3_ because the *c*-factor is increased to an optimized value. Here, we discuss that such increasing and optimization are necessary for the defective spinel γ-Al_2_O_3_ compound due to its existed aluminum vacancies, as aforementioned in the ‘Crystal structure’ subsection. To do this, it is useful to make clear the role of the *c*-factor in the mBJ method. To this end, we reformulate the mBJ exchange potential [7] in an appropriate form as follows:

(1) $$v_{x,\sigma }^{\mathrm{mBJ}}\left(\vec{r}\right)=c\left(v_{x,\sigma }^{\mathrm{BJ}}\left(\vec{r}\right)\frac{1}{\pi }\sqrt{\frac{10t_{\sigma }\left(\vec{r}\right)}{3\rho_{\sigma }\left(\vec{r}\right)}}\right)-\frac{1}{\pi }\sqrt{\frac{10t_{\sigma }\left(\vec{r}\right)}{3\rho_{\sigma }\left(\vec{r}\right)}}\text{,}$$

where *c*, *v*_*x*,*σ*_^*mBJ*^, *t*_*σ*_, and *ρ*_*σ*_ are the *c*-factor, multiplicative Becke and Johnson potential [9], kinetic energy density, and electron charge density, respectively. The *c*-factor linearly depends on the square root of the average of $\frac{\left|\nabla \rho_{\sigma }\right|}{\rho_{\sigma }}$ as follows [7]:

(2) $$c=1.023\sqrt{\frac{1}{\Omega }\int \frac{\left|\nabla ,\rho_{\sigma },\left(\vec{r}\right)\right|}{\rho_{\sigma }\left(\vec{r}\right)}\vec{d}r'}-0.012\text{,}$$

where the two 1.023 $\sqrt{bohr}$ and −0.012 (dimensionless) values were fitted by Tran and Blaha [7] in order to reproduce the experimental bandgaps of several compounds. From Equation 1, it is clear that if *c* = 1, then we have, $v_{x,\sigma }^{\mathrm{mBJ}}\left(\vec{r}\right)=v_{x,\sigma }^{\mathrm{BJ}}\left(\vec{r}\right)$ which is consistent with that of [7]. The *c*-factor in many cases can be well reproduced within a self-consistent calculation by the self-consistently converged charge density, *ρ*_*σ*_. However, for the γ-Al_2_O_3_ compound, the self-consistently reproduced *c*-factor is not calculated to be so large to be used for predicting the bandgap close to the experimental value. The *c*-factor may not be large enough for our case because the electron charge density for the defective spinel ${□}_{2\frac{2}{3}}Al_{21\frac{1}{3}}$O_32_ around the Al vacancy, as indicated by the empty box (□), is very small. Therefore, due to the vacancy, the *c*-factor which linearly depends on the square root of the average of $\frac{\left|\nabla \rho_{\sigma }\right|}{\rho_{\sigma }}$ is self-consistently calculated by Equation 2 to be smaller than an actual and necessary *c* value for reproducing the bandgap of the γ-Al_2_O_3_. Hence, we have increased the *c*-factor to overcome the low electron charge density that originated from the vacancy for our case. It is worth to note that, in practice, the *c*-factor cannot be unlimitedly increased. Thus, the experimental bandgap cannot be always exactly reproduced by increasing the *c*-factor in the current version of the mBJ method for every case without considering the electron charge density variation and bonding nature of the case. Indeed, there can be a critical value for the *c*-factor, as reported in [10]. For larger *c* values than the critical *c* value, the bandgap may be drastically decreased to a meaningless value far from the experiment. This shows that the mBJ potential may result in an incorrect electron charge density, if one uses a very large *c*-factor. The latter point was perfectly demonstrated [10] by calculating the electric field gradient, as an extremely sensitive physical quantity to the shape of the valence electron charge density, versus the *c*-factor for the Cu_2_O compound. Consequently, the *c*-factor, as a measure for the electron charge density, should be carefully examined and optimized at least for those cases having low electron charge density. The low charge density may originate from some hollow spaces due to the vacancies, as what existed in the γ-Al_2_O_3_ case, or from weak van der Waals bonds, as what existed in the fcc-C_60_ fullerite. Therefore, from the above discussion, we would, as a corollary result, anticipate that the *c*-factor may not be also well produced by the regular mBJ method for the case of fcc-C_60_. Thereby, one needs to first optimize the *c*-factor for this case as well. The latter anticipation needs more elaboration which is out of the scope of this work.

### Charge density

**Figure 4:**
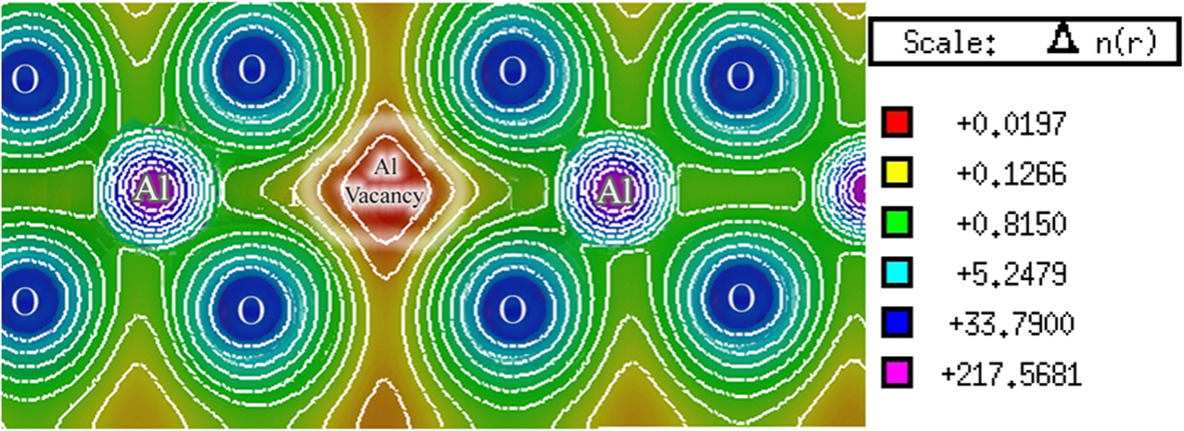
**Electron charge density of γ-Al**_**2**_**O**_**3**_**.** Electron charge density indicating the position of the aluminum vacancy. This result is obtained by setting the optimized *c*-factor, i.e., *c* = 1.8, in Equation 3.

A graphical description of the spatial features of the electronic structure has been presented by means of the electronic density [14]. Figure 4 presents the electronic charge density graph for the defective spinel structure of γ-Al_2_O_3_ which is calculated using the mBJ exchange potential. Clearly, the electronic cloud is mostly distributed around the oxygen nuclei. Moreover, in the Al-O inter-nuclear line, the electronic cloud is polarized toward the aluminum atoms. Neither the Al-Al nor O-O inter-nuclear lines show a polarized electronic cloud. This indicates that a highly polar bond is formed in the Al-O line, in γ-Al_2_O_3_, as expected from a large electronegativity difference between Al and O atoms. These results are consistent with previous reports (e.g., [6]). It should be pointed out that electron depletion is observed at the aluminum vacancy sites in Figure 4. Observation of such an electron depleted region is expected because a stoichiometric γ-Al_2_O_3_ structure itself is an electrically neutral solid material, and inclusion of an ionized aluminum vacancy (e.g., VAl''' point defects) makes the electric charges unbalanced. Moreover, the stoichiometric γ-Al_2_O_3_ structure is a perfect insulator, and formation of charge-free carriers is not anticipated from our calculation. In addition, it is observed in Figure 4 that distribution of the valence charge density is not uniform around different Al lattice sites. These results do not completely confirm the previous results that distribution of the valence charge density is uniform around the Al lattice sites, regardless if they are occupied or vacant [14]. We believe that our findings may be a little bit in better agreement with the γ-Al_2_O_3_ material because introduction of a vacancy affects the aluminum-oxygen interactions and therefore slightly disturbs the charge density distribution.

Figure 4 qualitatively shows that the Al atoms located at the octahedral positions, in contrast with the oxygen atoms, are covalently bonded to the O atoms. Al atoms are far from each other and thereby cannot be strongly bonded to each other. These observations are in agreement with the quantitative ionic charge analysis [14]. Therefore, the Al-O constitutes the most important bonding in the γ-Al_2_O_3_ crystal. This implies that if the electron charge density of the γ-Al_2_O_3_ crystal is subtracted from the superposition of its atomic densities, the Al-Al and O-O charge densities can almost cancel each other. This is consistent with the difference between the electron charge density of the γ-Al_2_O_3_ crystal and the superposition of its atomic densities [14]. There are two Al vacancies in the supercell, as stated in the ‘Crystal structure’ subsection and as shown in Figure 1, and two positions with two different point groups for placing them, octahedral (O) and tetrahedral (T) positions. Either both of the Al vacancies can be positioned in the octahedral (tetrahedral) and OO (TT) sites or one in the O site and the other in the T site, OT, as considered in [5]. Depending on the vacancies positions, 14 nonequivalent, 4 OO, 6 TO, and 4 TT configurations can be constructed for the system [5]. Total energy DFT calculations were performed using the ultrasoft Vanderbilt pseudopotential as embodied in the VASP code for these 14 configurations [5]. The minimum energy was found for an OO configuration, followed by an OT, and then a TT configuration with energy differences of 0.160 eV/(Al_2_O_3_) and 0.137 eV/(Al_2_O_3_) [5]. These small energy differences show that the total energy is not completely indifferent to the Al positions. This verifies that the most crystal bonding energies originate from Al-Al bonds. However, by neglecting these small energy differences, we would state that the total energy is almost insensitive to the Al positions, which is what would explain the disorder in the Al sublattice in agreement with [14]. Equation 1 can be equivalently rewritten in terms of Becke-Roussel (BR) exchange potential [15] as follows [7,10]:

(3) $$v_{x,\sigma }^{\mathrm{mBJ}}\left(\vec{r}\right)=cv_{x,\sigma }^{\mathrm{BR}}\left(\vec{r}\right)+\left(3c-2\right)\frac{1}{\pi }\sqrt{\frac{5}{6}}\sqrt{\frac{t\sigma \left(\vec{r}\right)}{\rho_{\sigma }\left(\vec{r}\right)}}$$

**Figure 5:**
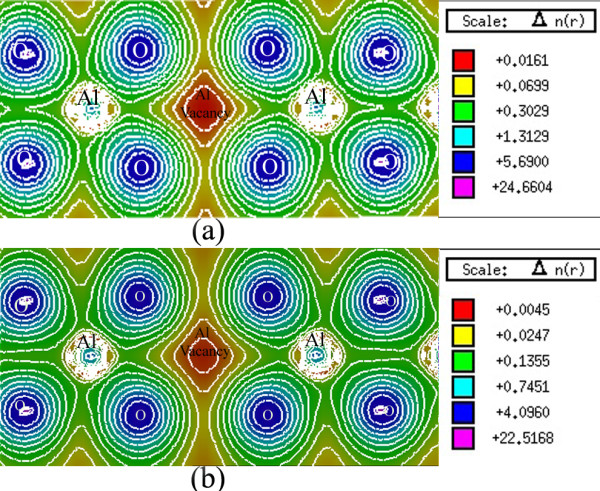
**BR and**tρ**terms corresponded to the electron charge density of γ-Al**_**2**_**O**_**3**_**.** (**a**) BR and (**b**) tρ terms corresponded to the total electron charge density, as given in Figure 4, indicating the position of the aluminum vacancy for γ-Al_2_O_3_. BR term is obtained from Equation 3 by setting $c=\frac{2}{3}\text{,}$ and tρ is obtained from Equation 3 by setting *c* = 0.

**Figure 6:**
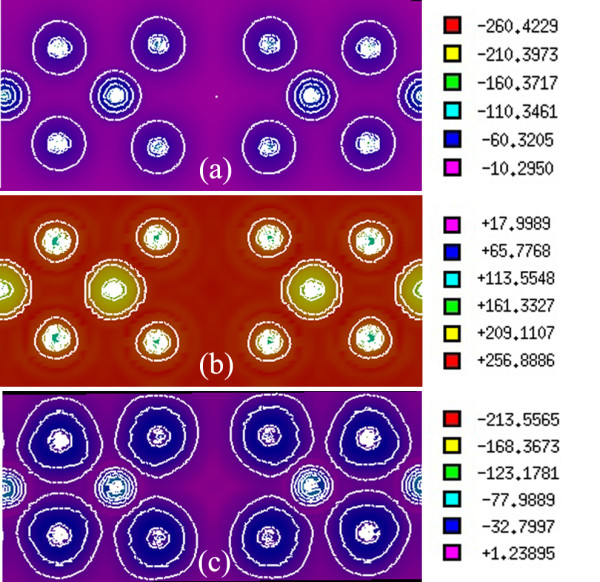
**Contour plots for**$v_{x,\sigma }^{\mathrm{BR}}\left(\vec{r}\right)$**and**$\sqrt{\frac{t_{\sigma }\left(\vec{r}\right)}{\rho_{\sigma }\left(\vec{r}\right)}}$**contributions of the total**$v_{x,\sigma }^{\mathrm{mBJ}}\left(\vec{r}\right)$**exchange potential of γ-Al**_**2**_**O**_**3**_**.** Two-dimensional plots for (**a**) $v_{x,\sigma }^{\mathrm{BR}}\left(\vec{r}\right)$ and (**b**) $\sqrt{\frac{t_{\sigma }\left(\vec{r}\right)}{\rho_{\sigma }\left(\vec{r}\right)}}$ contributions of (**c**) the total $v_{x,\sigma }^{\mathrm{mBJ}}\left(\vec{r}\right)$ exchange potential for γ-Al_2_O_3_. $v_{x,\sigma }^{\mathrm{BR}}\left(\vec{r}\right)$ is obtained from Equation 3 by setting $c=\frac{2}{3}\text{,}$ and tρ is obtained from Equation 3 by setting *c* = 0. $v_{x,\sigma }^{\mathrm{mBJ}}\left(\vec{r}\right)$ is obtained from Equation 3 using the optimized *c*-factor, i.e., *c* = 1.8.

**Figure 7:**
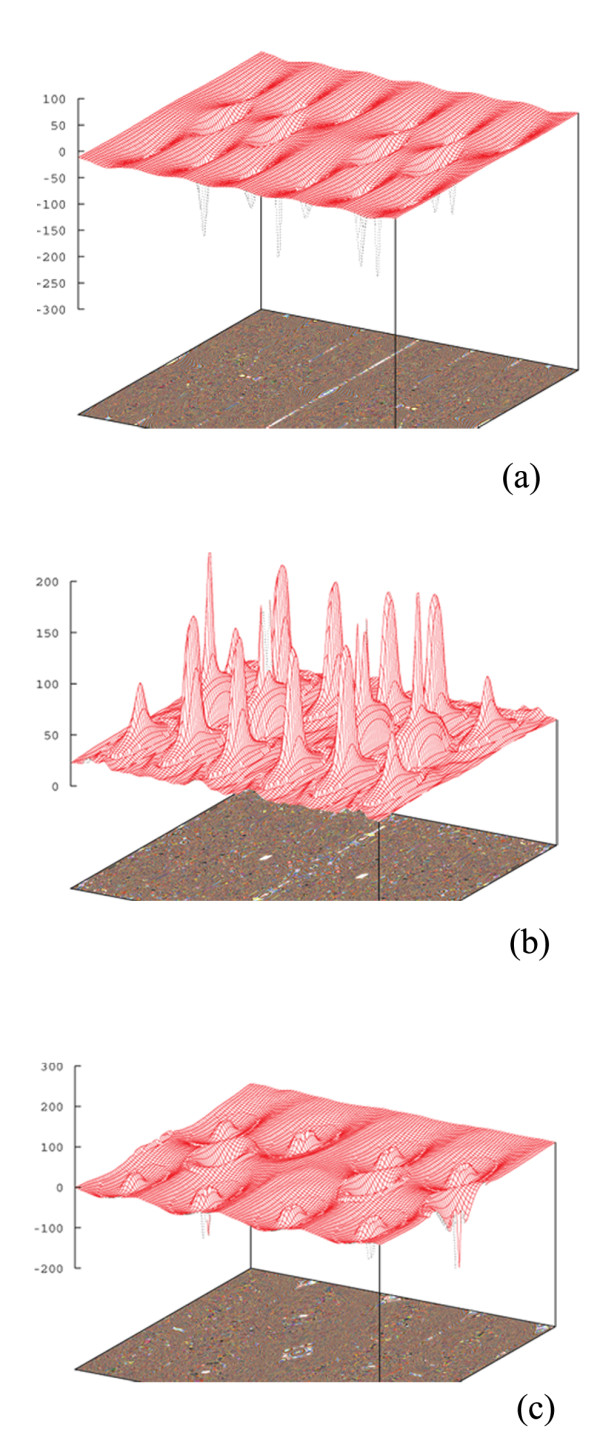
**Three-dimensional plots for**$v_{x,\sigma }^{\mathrm{BR}}\left(\vec{r}\right)$**and**$\sqrt{\frac{t_{\sigma }\left(\vec{r}\right)}{\rho_{\sigma }\left(\vec{r}\right)}}$**contributions of the total**$v_{x,\sigma }^{\mathrm{mBJ}}\left(\vec{r}\right)$**exchange potential of γ-Al**_**2**_**O**_**3**_**.** Three-dimensional plots for (**a**) $v_{x,\sigma }^{\mathrm{BR}}\left(\vec{r}\right)$ and (**b**) $\sqrt{\frac{t_{\sigma }\left(\vec{r}\right)}{\rho_{\sigma }\left(\vec{r}\right)}}$ contributions of (**c**) the total $v_{x,\sigma }^{\mathrm{mBJ}}\left(\vec{r}\right)$ exchange potential for γ-Al_2_O_3_. $v_{x,\sigma }^{\mathrm{BR}}\left(\vec{r}\right)$ is obtained from Equation 3 by setting $c=\frac{2}{3}\text{,}$ and tρ is obtained from Equation 3 by setting *c* = 0. $v_{x,\sigma }^{\mathrm{mBJ}}\left(\vec{r}\right)$ is obtained from Equation 3 using the optimized *c*-factor, i.e., *c* = 1.8.

The BR exchange potential tries to model the Coulomb potential created by the exchange hole and approaches asymptotically to the exact Kohn-Sham exchange potential [10]. The mBJ potential as given in Equation (3) consists of two terms - the BR term and the $\sqrt{t/\rho }$ term [10]. In addition to the total electron charge density, we have calculated the electron charge density generated only by the BR term, as shown in Figure 5a. The BR term is calculated by setting *c* = 2/3 in Equation 3. Furthermore, we have calculated the electron charge density generated only by the second term, $\sqrt{t/\rho }$ term, as shown in Figure 5b. The $\sqrt{t/\rho }$ term is calculated by setting *c* = 0 in Equation 3. The BR charge density, as shown in Figure 5a, is almost spherical, while the charge density is aspherical for the $\sqrt{t/\rho }$ term, as can be seen in Figure 5b. This result is in agreement with [10]. Exchange potentials that corresponded to the BR and $\sqrt{t/\rho }$ terms are similarly calculated. The $v_{x,\sigma }^{\mathrm{BR}}\left(\vec{r}\right)$, $\sqrt{\frac{t_{\sigma }\left(\vec{r}\right)}{\rho_{\sigma }\left(\vec{r}\right)}}$, and $v_{x,\sigma }^{\mathrm{mBJ}}\left(\vec{r}\right)$ exchange potentials are given in two-dimensional plots, as shown in Figure 6a,b,c, and in 3D plots, as shown in Figure 7a,b,c, respectively. The results show that the $v_{x,\sigma }^{\mathrm{BR}}\left(\vec{r}\right)$ is attractive (negative), while $\sqrt{\frac{t_{\sigma }\left(\vec{r}\right)}{\rho_{\sigma }\left(\vec{r}\right)}}$ is repulsive (positive) which is in complete accord with [10]. The total exchange potential, $v_{x,\sigma }^{\mathrm{mBJ}}\left(\vec{r}\right)$, (Figure 6c) can be obtained by applying Equation 3 on the $v_{x,\sigma }^{\mathrm{mBJ}}\left(\vec{r}\right)$ (Figure 6a), and $\sqrt{\frac{t_{\sigma }\left(\vec{r}\right)}{\rho_{\sigma }\left(\vec{r}\right)}}$ (Figure 6b) contributions for our optimized *c*-factor, i.e., *c* = 1.8. For example, for the red regions of Figure 6a,b,c, we can verify the latter point, *viz.*, $213.5565≅1.8\times \left(-260.4229\right)+\left(3\times 1.8-2\right)\times \frac{1}{\pi }\times \sqrt{\frac{5}{6}}\times 256.8886=-214.9687$. Therefore, it is now clear that the bandgap should be increased by increasing the *c*-factor: the signs of the BR and $\sqrt{t/\rho }$ terms are opposite, and BR is weighted by *c*, whereas $\sqrt{t/\rho }$ is weighted by 3*c*[10]. The repulsive potential,$\sqrt{t/\rho }$ is proportional to $\sqrt{1/\rho }$. Therefore, the $\sqrt{t/\rho }$ term in the mBJ potential will be of significant importance if the electron charge density is low. For our case, charge density is low in the regions near the vacancies. Thus, contribution of the repulsive term is important for the Al_2_O_3_ case, as can be seen in Figure 6b, and should not be neglected.

## Conclusions

We have successfully simulated the γ-Al_2_O_3_ compound based on the DFT method using the full potential augmented plan waves plus local orbital method, as embodied in the WIEN2k code, and applied the mBJ exchange potential on this system to predict its bandgap more precisely. We found that the Al-O has a highly polar bond in this compound which is consistent with previous reports. We showed that distribution of the valence charge density is not uniform around different Al lattice sites. Besides, the calculated partial DOSs indicated that majority of the valence electronic charges correspond to the p orbitals of oxygen atoms which are consistent with those of previous reports. However, we showed that different oxygen atoms have different contributions in the valence electronic charges. Contributions of oxygen atoms which are closer to the vacancies in γ-Al_2_O_3_ dominate. A charge-free region is observed at the aluminum vacancy site which confirms the charge neutrality and insulating behavior of the stoichiometric γ–Al_2_O_3_ structure. Our result shows that mBJ can significantly improve the electronic structure of the system if a suitable *c*-factor is used. A direct bandgap of 8.02 eV, which is very close to the experimentally measured value of 8.7 eV, was obtained at the Γ point by adjusting the *c*-factor internally to a value of 1.8. This *c*-factor value can be utilized for the correct estimation of the electronic and optical properties of γ-Al_2_O_3_ compound based on the full potential augmented plan waves plus local orbital DFT method using the mBJ exchange potential.

## Competing interests

The authors declare that they have no competing interests.

## Authors' contributions

MY performed the calculations and analyzed the results, as well as wrote the first draft of the manuscript. SJA supervised the computational part of the work, proposed to apply the regular mBJ method, gave the idea on how to apply and to perform the non-regular mBJ calculations to improve the result even better than the expensive quasiparticle Green function method, as well as wrote the final version of the manuscript and analyzed the results. AN, as an experimentalist, proposed working on the γ-Al_2_O_3_ due to its important technological applications, supervised the experimental part of the work, as well as drafted the manuscript and analyzed the results. MG participated in performing the regular mBJ calculations and experimental part of the work. MG also started the experimental and theoretical works at their first stages and opened the way for MY, who is continuing and extending the experimental and theoretical parts of the work. MR helped us to perform calculations needed for revising the manuscript. All authors read and approved the final manuscript.

## Authors' information

SJA has a PhD in Computational Condensed Matter Physics. AN has a PhD in Experimental Condensed Matter Physics. MY, MG, and MR are MS students.

## References

[B1] Halasz I Brenner A Shelef M Catalytic reduction of nitric oxide on PdO—MoO_3_/γ-Al_2_O_3_ Applied Catalysis B: Environmental 1993 2 131 146 10.1016/0926-3373(93)80031-8

[B2] O'Connor BH Li DY Gan BK Latella B Carter J Time-resolved studies of alumina ceramics processing with neutron diffraction and synchrotron radiation data Advances in X-ray Analysis 1999 41 659 667

[B3] Chou TC Nieh TG Interface-controlled phase transformation and abnormal grain growth of α-Al_2_O_3_ in thin γ-alumina films Thin Solid Films 1992 221 89 97 10.1016/0040-6090(92)90800-Q

[B4] Molas G Bocquet M Vianello E Perniola L Grampeix H Colonna JP Masarotto L Martin F Brianceau P Gély M Bongiorno C Lombardo S Pananakakis G Ghibaudo G De Salvo B Reliability of charge trapping memories with high-k control dielectrics (invited paper) Microelectronic Engineering 2009 86 1796 1803 10.1016/j.mee.2009.03.083

[B5] Gutiérrez G Taga A Johansson B Theoretical structure determination of γ-Al2O3 Physical Review B 2001 65 012101

[B6] Pinto HP Nieminen RM Elliott SD *Ab initio* study of γ-Al_2_O_3_ surfaces Physical Review B 2004 70 125402

[B7] Tran F Blaha P Accurate band gaps of semiconductors and insulators with a semilocal exchange-correlation potential Physical Review Letters 2009 102 226401 19658882 10.1103/PhysRevLett.102.226401

[B8] Blaha P Schwarz K Madsen GKH Kvasnicka D Luitz J WIEN2K: An Augmented Plane Wave plus Local Orbitals Program for Calculating Crystal Properties 2001 Vienna: Vienna University of Technology

[B9] Becke AD Johnson ER A simple effective potential for exchange Journal of Chemical Physics 2006 124 221101 10.1063/1.2213970 16784253

[B10] Koller D Tran F Blaha P Merits and limits of the modified Becke-Johnson exchange potential Physical Review B 2011 83 195134

[B11] Verwey EJW Electrolytic conduction of a solid insulator at high fields The formation of the anodic oxide film on aluminium Physica 1935 2 1059 1063 10.1016/S0031-8914(35)90193-8

[B12] Ealet B Elyakhloufi MH Gillet E Ricci M Electronic and crystallographic structure of γ-alumina thin films Thin Solid Films 1994 250 92 100 10.1016/0040-6090(94)90171-6

[B13] Sankaran K Pourtois G Degraeve R Zahid MB Rignanese G-M Houdt JV First-principles modeling of intrinsic and extrinsic defects in γ-Al_2_O_3_ Appl Phys Lett 2010 97 212906 10.1063/1.3507385

[B14] Menéndez-Proupin E Gutiérrez G Electronic properties of bulk γ-Al_2_O_3_ Physical Review B 2005 72 035116

[B15] Becke AD Roussel ER Exchange holes in inhomogeneous systems: a coordinate-space model Physical Review A 1989 39 3761 10.1103/PhysRevA.39.3761 9901696

